# Neural Similarity and Synchrony among Friends

**DOI:** 10.3390/brainsci14060562

**Published:** 2024-05-30

**Authors:** Chao Ma, Yi Liu

**Affiliations:** 1 School of Psychology, Northeast Normal University, Changchun 130024, China; machao@nenu.edu.cn; 2 Jilin Provincial Key Laboratory of Cognitive Neuroscience and Brain Development, Changchun 130024, China

**Keywords:** neural similarity, neural synchrony, friendship, social distance, social interaction

## Abstract

Researchers have long recognized that friends tend to exhibit behaviors that are more similar to each other than to those of non-friends. In recent years, the concept of neural similarity or neural synchrony among friends has garnered significant attention. This body of research bifurcates into two primary areas of focus: the specificity of neural similarity among friends (vs. non-friends) and the situational factors that influence neural synchrony among friends. This review synthesizes the complex findings to date, highlighting consistencies and identifying gaps in the current understanding. It aims to provide a coherent overview of the nuanced interplay between social relationships and neural processes, offering valuable insights for future investigations in this field.

## 1. Introduction

In human society, individuals often choose their friends based on the principle of similarity, encapsulated by the adage “Birds of a feather flock together” [1]. Extensive research has shown that friends typically exhibit numerous similarities, not only in demographic characteristics such as age, gender, height, and race [2], but also in psychological characteristics like personality [3], attitudes [4], interests [5], and even biological characteristics such as genes [6]. In recent years, with the advancement of neuroscience, the neural similarities among friends have increasingly attracted attention. Specifically, in studies focusing on interactive situations, the neural similarities between two individuals were called neural synchrony to emphasize the interactive nature of the ongoing task.

According to Brent et al. [7], friends are defined as pairs of individuals engaging in bidirectional affiliative (nonaggressive and nonreproductive) interactions with sufficient frequency and consistency to distinguish them from non-friends. However, applying this phenotype-based definition to filter the literature about neural similarity/synchrony among friends proved challenging. Most studies on neural similarity/synchrony among friends rely on participants’ subjective self-reports to determine friendships, without qualitative measurements of the phenotype, such as affiliation or frequency of interactions. Therefore, we relied on the participants’ subjective reports of friendship and included all the literature that featuring friend dyads or participants within a friendship network in our review. We conducted our literature search using databases, i.e., Web of Science, Scopus, PubMed, and ProQuest, with keywords (“neural similarity” OR ”interpersonal neural synchronization” OR “brain synchronization” OR “interbrain synchronization” OR “interbrain synchrony” OR “Interindividual synchronization”) AND (“friends”). All the literature was meticulously checked, and 19 studies were included in our review (see Table 1).

This review first succinctly summarizes the research methods used to assess neural similarity and synchrony in prior studies, and then delve into the literature focusing on two primary research questions: (1) the specificity of neural similarity/synchrony among friends, and (2) the situational factors that influence neural synchrony among friends. Finally, we discuss the current findings, highlight the limitations, and propose future directions for research in this burgeoning field.

## 2. Methodology Used to Assess Neural Similarity and Synchrony

Existing studies have used functional near-infrared spectroscopy (fNIRS), functional magnetic resonance imaging (fMRI), and electroencephalographs (EEG) to record brain activities and calculate the inter-subject neural similarity or synchrony (INS) among friend dyads. EEG directly record an electrogram of the neuronal electrical activity, offering a high temporal resolution [27]. This allows for a detailed examination of the neural synchronization dynamics in both time and phase across various frequency bands [10,11]. However, EEG is more susceptible to environmental and physiological artifacts, and their spatial resolution is limited by the scalp-recorded signals [27]. In contrast, both fNIRS and fMRI quantify the ratio of oxygenated to deoxygenated hemoglobin, commonly referred to as the blood oxygenation level-dependent (BOLD) signal, to capture the neural activity within the brain [28,29]. Due to the inherently slow nature of the BOLD responses compared to the rapid neuronal electronic signals, both fMRI and fNIRS have lower temporal resolutions than EEG [30]. The distinction between fNIRS and fMRI lies in their methods of BOLD recording: fNIRS uses near-infrared light to detect changes in the cortical hemodynamic activity, while fMRI detects these changes by monitoring the electromagnetic signals emitted by hydrogen protons during imposed magnetic moment (spin) changes [29]. These different recording principles lead to significant distinctions in application. Firstly, fNIRS is limited by the penetration depth of near-infrared light, restricting its recording of the BOLD signals to the cortical regions [28]. Conversely, fMRI can capture BOLD signals from the entire brain with a relatively high spatial resolution [29]. Thus, fMRI is the preferable choice for studies requiring whole-brain exploration to identify neural similarity or synchrony among friends. Secondly, fNIRS exhibits a higher tolerance for motor artifacts compared to fMRI, making it commonly used in studies involving live social interactions due to its high ecological validity [31]. Considering the advantages and disadvantages of these techniques, researchers have adopted different methods to assess neural similarity and neural synchrony.

There are four types of INS calculations based on the brain activities recorded using fMRI. (1) Temporal INS: This is calculated as the correlations of the time series of the BOLD signals from the same brain region between subjects, describing the consistency of the time dynamics of the brain activities between two individuals. This method, known as traditional inter-subject correlation (ISC) analysis, uses the correlation coefficient to represent the degree of similarity [22,32]. While this index highlights the temporal dynamics of neural responses, it disregards voxel-based spatial information. Consequently, spatial INS has been introduced as an alternative index. (2) Spatial INS: This is calculated as the correlations between the averaged spatially distributed multi-voxel response patterns of the same region during a period (e.g., viewing a meaningful scene) between two individuals [12,32]. Although this method can capture voxel-based spatial patterns of activation across different time points, averaging the patterns from diverse time points may overlook some temporal details. (3) Spatio-temporal INS: This integrates the temporal dynamics and spatially distributed multi-voxel patterns. Firstly, the correlation or Euclidean distance between the multi-voxel response patterns for each time point are calculated to generate a TR x TR matrix (i.e., a pattern trajectory matrix) for each region. Then, the Pearson correlations or Euclidean distances between the vectorized pattern trajectory matrices of two individuals are calculated as the spatio-temporal INS of the same regions [12,32]. These three aforementioned methods focus on the similarity of neural responses within single regions. In addition, the functional connectivity between regions can also demonstrate neural similarities. (4) Functional connectomic INS: This is calculated as the Euclidean distances or correlation between the corresponding functional connectivity values between two brain regions or the whole-brain functional connectivity matrices of two individuals [13,20]. It overlooks the spatial information of single regions and emphasizes the similarity of the temporal dynamics between regions. These methods complement each other, each with distinct emphases.

For fNIRS, two types of INS indices are calculated. (1) Temporal INS: Similar to the temporal INS in fMRI, it is calculated as the correlation coefficients between the time series of the BOLD signals of the brain regions from two individuals [9,16,23]. (2) Wavelet Transform Coherence (WTC): This method estimates a coherence coefficient between time series of each dyad as a function of frequency and time, reflecting both the time-related and frequency-related properties of the two time series [33]. Since studies using fNIRS often focus on neural synchrony during real social interactions such as cooperation and verbal communication, neural activities are always recorded through hyperscanning, i.e., the simultaneous recording of neural activities from dual brains during the live interaction [34]. This interactive nature of fNIRS recording allows for the calculation of time-aligned or time-lagged INS. For time-aligned INS, the synchronization is calculated using the temporally aligned brain activities of two individuals [21,25,26]. For time-lagged INS, the brain activity of one individual temporally lags behind that of the other [17,18], reflecting interpersonal predictive coding and delayed processing during social interactions [35]. Furthermore, INS can be calculated for the same brain regions [21,25,26] and also for different regions of the two individuals to describe the potential information flow between the two individuals during an interaction [17] (see discussion for details). Relative to neural similarity calculated with fMRI data, WTC incorporates the frequency information concerning the neural responses, a feature lacking in fMRI INS analyses. However, the relatively low spatial resolution of fNIRS precludes voxel-based spatial INS calculations. 

For EEG, three types of INS indices are calculated. (1) Temporal INS: This is calculated as the correlation coefficients between two time series of the EEG signals [8]. (2) Phase locking value (PLV): The PLV measures whether the EEG signals from the two interacting individuals are phase-locked across time [11,14,19]. (3) Circular Correlation Coefficient (CCorr): This is based on instantaneous phases and reflects the degree of covariation of the paired EEG signals, which is less susceptible in detecting spurious hyperconnections [15,36]. However, due to the low spatial resolution of EEG, conclusions regarding the specific brain regions involved in these studies remain speculative.

## 3. Specificity of Neural Similarity and Synchrony among Friends

Based on the above indices, there are two lines of research focused on the specificity of the neural similarity or neural synchrony between friends. One is neural similarity in non-interactive situations and another is neural synchrony in interactive situations.

### 3.1. Neural Similarity in Non-Interactive Situations

This line of research mainly focused on investigating whether the establishment of friendships correlates with neural similarity between individuals. To this end, the researchers typically recruited participants from social groups and defined friendship based on social distance between two individuals within a social network. The fundamental assumption posits that, within a social group, individuals are free to select their friends during the initial stages of group formation, possibly guided by the principle of similarity. Consequently, social distance serves as an effective proxy for friendship, reflecting the underlying neural similarity. In this measurement of friendship, the participants were asked to identify others in a social group as friends, with each individual represented as a node. If a pair of individuals mutually identified one another as friends, they were connected with an edge. Social distance was assessed as the shortest length of the connections between any two individuals based on the established social network. A social distance of one indicates mutual friendship, while a distance greater than one indicates they are not direct friends. All participants were from the same social network, and their brain activity was recorded individually without any interactive tasks, such as watching videos [8,12,22] or rest [13,20]. 

For instance, Parkinson et al. [22] recruited participants from an academic cohort and asked them to name their friends. The neural activities were recorded using fMRI while participants passively watched a series of video clips with various contents. The conventional ISC method based on Pearson correlations between the time series of their fMRI responses was employed to assess the inter-subject neural similarity. The results showed that neural similarity decreases with increasing social distance in the real-world social network. Specifically, the brain regions that showed significant neural similarity among friends was in the cortical regions related to attention (i.e., the superior and inferior parietal lobe) and also in the subcortical regions related to motivation and affective processing (i.e., the nucleus accumbens, amygdala, putamen, and caudate nucleus). These results suggest that social networks may be associated with similarities in how individuals attend to, interpret, and emotionally react to the world around them. Furthermore, given that conventional ISC solely captures information about similarity in voxel-averaged neural temporal dynamics while disregarding finer-grained, spatially distributed response topographies, Hyon et al. [12] reanalyzed the data from Parkinson et al. [22], calculating the inter-subject similarity of the averaged spatial multi-voxel response patterns for each event and the temporal trajectory of the multi-voxel response patterns. The results showed that the spatial INS (averaged for each event) was not associated with social distance. In contrast, the spatio-temporal INS in the dorsal attention network (specifically, in the superior parietal lobule) was found to be associated with social distance, consistent with Parkinson et al. [22]. In addition to the dyad-level friendship, Baek et al. [8] used in-degree centrality to represent an individual’s proximity within the social group, meaning individuals with a higher in-degree centrality in a social network are considered friends by more others. The results showed that these more central individuals had neural responses similar to the averaged neural responses of all other members and to each other in the regions of the default mode network (i.e., the bilateral dorsomedial prefrontal cortex and precuneus), whereas the less central individuals exhibited more variable responses. 

Furthermore, researchers have shown interest in assessing the interpersonal functional connectivity similarity at rest. McNabb et al. [20] and Hyon et al. [13] almost at the same time examined school-aged girls and adults, respectively, using fMRI and employed functional connectomic INS as an index of neural similarity. However, their findings yielded inconsistencies. McNabb et al. [20] found no associations between neural similarity and social network proximity, whereas Hyon et al. [13] demonstrated a positive correlation between the similarity in functional connectomes and social network proximity, particularly within the default mode network. This positive correlation was attributed to sustained and intensive interactions among friends, leading to convergence in cognitive, emotional, and behavioral styles during rest. It is important to note that the participants in Hyon et al. [13] were from one village and included spouses, not just friends, but the authors named their social network as a friendship network.

Collectively, with regard to the specificity of neural similarity among friends as defined by social networks, the extant findings consistently indicate a positive relationship between social network proximity and neural similarity elicited by exogenous stimuli (i.e., video watching), particularly in the dorsal attention network [12,22] and the default mode network [8]. However, further exploration is warranted regarding the specificity of neural similarity among friends during rest. It is important to note that the association between friendship and neural similarity can be interpreted in two ways. Firstly, individuals with greater neural similarity may respond to the environment similarly, thus fostering friendship formation. In short, it is the shared neural responses that engender friendships. Alternatively, neural similarity among friends may be shaped by frequent interactions and shared experiences. However, due to the cross-sectional nature of these studies, it remains challenging to ascertain whether neural similarity serves as a cause or a consequence of friendship.

### 3.2. Neural Synchrony in Interactive Situations

In interactive situations, participants engage in interpersonal tasks simultaneously, while their brain activity is recorded using hyperscanning neuroimaging techniques. The control condition typically includes stranger dyads performing identical tasks to friend dyads. Although it is hypothesized that neural synchrony is more pronounced among friends compared to strangers, the empirical findings have not consistently confirmed this specificity.

Specifically, Pan et al. [21] explored neural synchrony during a cooperative task (i.e., pressing a key as simultaneously as possible upon seeing a signal), during which the brain activity in the right superior frontal cortex was recorded with fNIRS. The results showed that the inter-subject neural synchrony among strangers was comparable to that among friends. Similarly, Djalovski et al. [11] measured brain activities using EEG, while the participants cooperated to draw predefined abstract images. Using the PLV as an index, no significant differences were found in neural synchrony between friends and strangers. Conversely, under non-cooperative conditions, Luft et al. [19] employed an independent time reproduction task and demonstrated greater interpersonal neural synchrony among friends during eye contact, measured using the average network strength of the PLV of the gamma band. Song et al. [23] used the joint Simon task, where individuals performed independently but the outcomes depended on joint actions, finding that neural synchrony across the dorsolateral and medial parts of the prefrontal cortex was stronger for friends than for strangers. While under a competitive task (i.e., pressing a key faster than their partners), Jia et al. [14] found that neural synchrony (the PLV in the theta band) was stronger for friends than strangers within an 800–1000 ms window, yet this pattern reversed in the 400–600 ms window.

To date, the evidence concerning the specificity of interpersonal neural synchrony among friends in interactive contexts remains complex. One plausible explanation is that the neural synchrony observed during cooperative tasks is driven more by the task demands rather than the interpersonal relationship itself. Even interactions between strangers can enhance coordination, which in turn supports neural synchrony, diminishing the distinctions typically observed with friends [11,21]. In contrast, the stronger neural synchrony observed among friends in non-cooperative tasks may stem from specific social bindings, such as affective engagement or familiarity, which are not as pronounced between strangers [19,23]. However, this inference needs further evidence in the future.

In summary, in contrast to the nuanced findings regarding neural synchrony during interactive tasks, the specificity of neural similarity among friends in non-interactive contexts appears to be robust. These findings suggest that neural similarities observed with natural stimuli may more accurately reflect friendships, potentially due to prolonged shared experiences or because individuals with similar neural responses tend to form friendships. However, it seems that the task dependency of neural synchrony makes the specificity of friendships seem elusive. 

## 4. Situational Modulations on Neural Synchrony among Friends

While friends may not consistently exhibit robust specific neural synchrony compared to strangers during interactive tasks, research suggests that certain conditions can enhance neural synchrony among friends.

### 4.1. Neural Synchrony during Free Communication

Spiegelhalder et al. [24] asked friend dyads to discuss autobiographical life events, alternating between one participant describing a life event while the other listened, and vice versa, during fMRI hyperscanning. The results revealed that the neural activity in the speech production areas of the speaker was synchronized with that in the listener’s auditory cortex and posterior cingulate cortex. This inter-brain neural synchrony between distinct regions implies a reciprocal mechanism of social interaction. Moreover, such synchrony was absent in dyads not engaged in actual conversation. Similarly, Long et al. [17], using fNIRS, demonstrated that face-to-face verbal communication among heterosexual friends induced time-lagged neural synchrony, notably from the anterior temporal lobe (ATL) in women to the temporoparietal junction (TPJ) and sensorimotor cortex in men, highlighting the interpersonal interaction between speech production and mentalizing systems during conversation. These findings underscore the induction of interpersonal neural synchrony through active verbal communication.

Further, Long et al. [18] also found that discussing supportive topics increased the women-led time-lagged neural synchrony between the sensorimotor cortex of friend dyads more than discussions centered around conflict. In another study, Liu et al. [16] explored neural synchrony during interpersonal emotion regulation employing different regulation strategies (i.e., cognitive reappraisal and expressive suppression). They found that cognitive reappraisal heightened synchrony between the prefrontal and temporal areas during the sharing stage, while expressive suppression increased synchrony in the prefrontal cortex during the regulation stage. These findings underscore the influence of communication content, such as the valence or regulatory strategies employed, on neural synchrony. However, these explorations of the situational modulators on neural synchrony represent just the beginning, and further converging evidence is required.

### 4.2. Neural Synchrony during Goal-Directed Tasks

In addition to free communication, numerous studies have delved into neural synchrony during specific goal-directed tasks, where dyads may need to cooperate or perform tasks independently to achieve their goals. It can be inferred that positive social cues can enhance the inter-brain neural synchrony between friends. 

For instance, a “gift effect” on neural synchrony was demonstrated. That is, one participant was asked to previously prepare a gift and donate it to his/her partner during the experiment. After that, the inter-brain neural synchrony during an attentional cooperative task indexed by oxygenated hemoglobin [9] and the correlation coefficients of EEG signals [10] in the dorsolateral prefrontal cortex increased (compared with the neural synchrony before the gift exchange), accompanied by enhanced perceived cooperation and cognitive performance. Moreover, regarding eye contact as an inherently positive social cue between friends, Luft et al. [19] found that, compared to no contact, eye contact between friends could augment inter-brain neural synchrony in the gamma frequency band during an independent time reproduction task. Additionally, Jia et al. [15] demonstrated that not only eye contact but also hand contact could increase inter-brain neural synchrony between friends in a motor imagery task. Additionally, neural synchrony between friends could be enhanced during cooperative problem-solving (i.e., a tangram puzzle task), compared to solving problems independently [25]. These findings suggest that the interventions that reinforce social bonds (i.e., gift exchange, eye/hand contact, or cooperation) could effectively heighten inter-brain neural synchrony during goal-directed tasks. As a result, when viewing the outcome of a risk-taking cooperation task, negative feedback (vs. positive feedback) induced stronger neural synchrony between friends, which may arouse stronger emotion connections between friends [26].

In summary, communicative cues signaling positive social interaction can significantly increase neural synchrony between friends, both during free communication and goal-directed tasks. 

## 5. Discussion

### 5.1. Stimuli and Tasks Used in Previous Studies

With the exception of studies investigating neural similarity of the resting state [13,20], all other investigations on neural similarity among friends used a combination of various videos (e.g., speeches, comedies, weddings, etc.) as a continuous video stimulus. Based on this, the specificity of neural similarity among friends was observed in attention-related regions, attributed to friends attending to the world in similar ways [12,22]. Notably, it is possible that the similarity in attention-related neural responses may stem from similar levels of engagement to various videos, reflecting similar personal interests to these videos. Therefore, besides examining attention-related neural responses across different video types, it is imperative to explore whether friends demonstrate similar neural processing in specific domains, such as social information or emotional content. Future research could elucidate the context dependency of neural similarity between friends, which holds practical implications for daily interactions and friend selection.

As for studies on neural synchrony in interactive situations, one line of research employed free communication tasks [17,18,24]. The turn-taking nature of verbal conversation highlights both time-aligned neural synchrony across different brain regions [16,24] and time-lagged neural synchrony within the same brain region [17,18] in the two communicating brains. However, the high ecological validity of free conversation also entails low experimental control. Addressing this gap, another line of research used interactive tasks under contexts of cooperation [9,10,11,21,25], competition [14], or individual task performance [15,19,25]. Although these studies identified neural synchrony between friends, the diverse types of interactions (i.e., cooperation, competition, and independent tasks) complicate direct comparisons. A systematic comparison of these tasks to investigate whether the interaction type could serve as a situational modulator on neural synchrony among friends is warranted in future research.

### 5.2. Brain Regions and Their Functional Meanings

**Figure 1:**
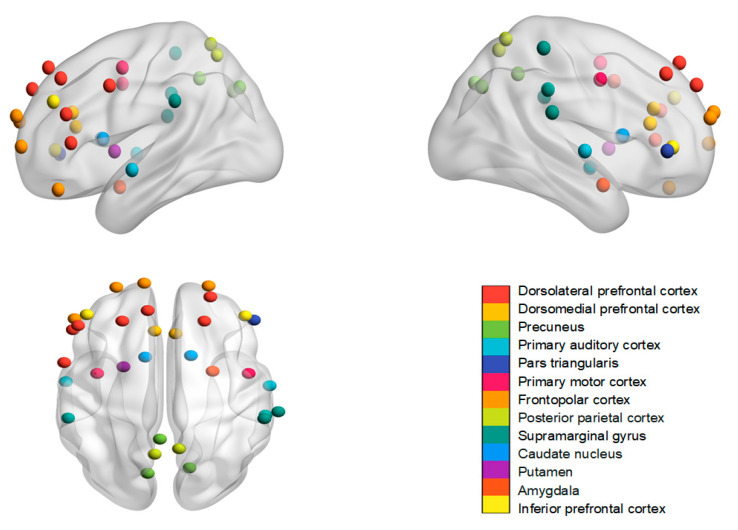
Overview of brain regions mentioned in the existing fNIRS and fMRI studies.

At first, we summarized the brain regions mentioned in the fNIRS and fMRI studies and the frequency bands used in the EEG studies. In the fMRI research, neural similarity or synchrony among friends was identified in the dorsomedial prefrontal cortex and precuneus [8]; the posterior parietal cortex [12]; the superior and inferior parietal lobe, nucleus accumbens, amygdala, putamen, and caudate nucleus [22]; and the speech production areas, auditory cortex and posterior cingulate cortex [24]. The fNIRS studies have focused on various brain regions including the dorsolateral prefrontal cortex [9,16,23,25,26]; the medial prefrontal cortex [23]; the inferior prefrontal gyrus and frontopolar cortex [26]; the temporoparietal junction [17,25]; the sensorimotor cortex [17,18]; the anterior temporal lobe [17]; the supramarginal gyrus [16,25]; and the right pars triangularis [25] (Figure 1). In the EEG research, neural synchrony was predominantly observed in the gamma band [19], delta band [10], and theta band [10,14]. Due to the limited spatial resolution of EEG, most studies did not label their findings with specific brain regions. In addition, two studies focused on neural similarity/synchrony based on the entire brain network, without targeting specific regions or electrodes [13,20].

It can be seen that the brain regions demonstrating neural similarity or synchrony among friends exhibit considerable variation in location and are heavily contingent upon the task at hand. The interpretations regarding neural similarity and synchrony are rooted in the functions of these brain regions and the psychological processes engaged by the experimental task. For instance, the specificity of neural similarity observed among friends often manifests in regions associated with attention, which is explained by the shared manner in which friends attend to their surroundings [12,22]. Similarly, the connection between neural synchrony and behavioral performance, supporting the functional meaning of neural synchrony, remains unexplored. In addition, in studies of neural synchrony during live interactions, regions of interest are typically predefined before the fNIRS recording, based on the task requirements. For example, Long et al. [17] observed neural synchrony among friends in socially pertinent regions, such as the anterior temporal lobe (ATL) and temporoparietal junction (TPJ), responsible for understanding others’ mental states. In a related study, Long et al. [18] investigated the role of emotion in the dual-brain mechanisms of interpersonal communication, identifying neural synchrony in the sensorimotor cortex, which they associated with embodied emotional processes. However, due to the limitations inherent to fNIRS, different studies concentrate on different task-related regions, which impedes cross-study comparisons. 

Although these findings demonstrate the existence of neural similarity or synchrony among friends under specific tasks, the underlying psychological processes driving these phenomena remain unclear. The explanations for neural synchrony often rely on the presumed functions of the implicated brain regions, yet empirical support for these interpretations is limited. Furthermore, the causal relationship between neural similarity and social distance has yet to be established.

### 5.3. Distinctions between Neural Similarity and Neural Synchrony

Although the terms “neural similarity” and “neural synchrony” both refer to the correlations in the neural activities of dyads, they are applied in different contexts. “Neural synchrony” specifically pertains to the state-like inter-subject neural correlations observed during interactive tasks, where processes such as joint attention, shared emotions, mental state inference, and active mutual adaptation are engaged [16]. For example, interpersonal neural synchrony might manifest in the regions associated with language during discussions [24] and in the dorsolateral prefrontal cortex (dlPFC) during cooperative attentional tasks [9,10]. Hence, the brain regions exhibiting neural synchrony can vary depending on the nature of the interactive task and be modulated by situational factors. In contrast, “neural similarity” refers to correlations that are not contingent on interactive contexts, reflecting a tacit understanding between friends. Such trait-like neural similarity may be explained by shared responses to external stimuli or internal states among friends, leading to similar temporal and spatial patterns of brain activities [12,22].

Thus, neural similarity is typically time-aligned and focuses on the same brain regions among friends [8,12,22]. In contrast, neural synchrony can manifest as either time-aligned or time-lagged due to its interactive nature. Time-aligned INS involves the simultaneous brain activities of two individuals and indicates concurrent mentalizing or shared actions during interaction [9,10,16,21,23,24,25,26]. Time-lagged INS, conversely, reflects interpersonal predictive coding and the delayed response of one individual to another, such as in conversational dynamics where one person’s brain activity follows another’s [17,18]. Additionally, the interactive context of neural synchrony allows for the measurement of correlations between different brain regions based on the roles individuals play during interaction, such as between the language production areas of the speaker and the comprehension areas of the listener during turn-taking discussions [24]. This type of neural synchrony can capture the essence of communicative interaction and may indicate the motivation, involvement, or communication efficiency of the dyads [16]. 

### 5.4. Comparisons between Research Methods

Comparing research methodologies helps to reveal the distinct advantages and limitations of fNIRS, fMRI, and EEG, thus providing clarity about future directions. First, fNIRS allows for face-to-face communication and complex interactive tasks, such as cooperative drawing [11]. Studies focusing on neural synchrony during interactions typically employ this technology [17,18,21]. In contrast, fMRI restricts head movement and participants cannot see each other. Consequently, friend dyads in fMRI scanners cannot engage in face-to-face communication, which limits the demonstration of neural synchrony in the broader regions involved in live social interactions. In contrast, the studies examining neural similarity among friends that do not rely on live social interaction typically use fMRI to observe the whole brain during non-interactive tasks like video watching [12,13,22]. Second, fNIRS typically uses the WTC index to calculate INS, considering both time and frequency information [37]. However, to date, the neural similarity calculated using fMRI data has not considered the frequency information of the BOLD signals. In contrast, fMRI employs a more diverse array of methods for analyzing neural similarity. That is, traditional ISC analysis is used if only time information is considered [8,22]; multi-voxel pattern analysis is applicable if only spatial information is considered, benefiting from its high spatial resolution [12]; and analysis of time trajectory matrices is used for combining both time and space information [21]. Thus, fMRI-based analysis could offer a broader range of methods to address this issue. Third, EEG is sensitive to capture the differences in time dynamics due to its high temporal resolution. For example, Jia et al. [14] used EEG to assess neural synchrony and found that the difference between friends and strangers showed different patterns in different time windows (i.e., 400–600 ms vs. 800–1000 ms) during a simple button press task. Moreover, the PLV index specific to EEG reflects whether the neural responses from the two individuals were phase-locked across time, which is not used for fNIRS and fMRI.

In summary, to accentuate strengths and mitigate weaknesses, more innovative approaches that allow for live interaction during hyperscanning, such as using fMRI-compatible video cameras or well-controlled cooperative tasks in fMRI, are promising for detecting the neural synchrony in the whole brain as a mechanism of social interactions between friends. Alternatively, providing multimodal evidence from EEG, fNIRS and fMRI to complement each other would be more comprehensive for the characterization of interpersonal neural synchrony.

## 6. Limitations and Future Directions

Existing research on neural similarity and neural synchrony among friends has made significant progress, yet several limitations persist and numerous questions remain unexplored. Here are some critical considerations for future research:

(1) Confirming conclusions: The current findings suggest that the specificity of neural similarity when viewing videos is robust, whereas the specificity of neural synchrony during interactive tasks is elusive and dependent on interactive contexts. Future research needs to clarify the contextual moderators on the specificity of neural synchrony. For example, exploring the factors that differ between friends and strangers, such as empathy, can be beneficial in identifying the specificity of neural synchrony among friends. In addition, although positive social cues appear to promote neural synchrony, the evidence comes from various manipulations (e.g., eye contact and gift exchange) and under different tasks (e.g., tangram puzzle and time reproduction tasks). More comparable and converging evidence is needed.
(2) Verifying explanations: The current explanations for neural synchrony often rely on post hoc interpretations based on the experimental task or the common functions of the involved brain regions. Future studies could develop new interactive paradigms that precisely manipulate these situations to test which behavioral or psychological processes drive neural synchrony. Moreover, although the existing studies have established a link between neural similarity and friendship, the causal relationship remains unclear, due to the cross-sectional nature of most research. Longitudinal studies could help trace the developmental trajectory of neural similarity between friends, exploring whether this similarity exists before friendship formation and how it evolves over time.
(3) Standardizing definitions of friendship: The definition of ‘friend’ varies across studies. In studies focusing on neural similarity in non-interactive contexts, friends are defined within a specific social network [8,12,13,22], whereas in interactive studies friendship is self-reported without objective measures of closeness, such as duration or quality [9,10,11,14,15,16,17,18,19,21,23,24,25,26]. Moreover, the existing studies on the specificity of neural similarity or synchrony commonly use strangers rather than acquaintances as controls. Future research should clearly define friendship and consider incorporating measures of friendship quality like perceived closeness or mutual support, and replace the stranger control group with acquaintances to better capture the social attachment nature of friendships. In addition, future studies should compare the neural similarities between same-gender [9,16,24] and opposite-gender [11,17,18,21] friends to enhance the understanding of social relationships from a gender perspective.
(4) Integrating multiple research methods: Different technologies, i.e., fMRI, fNIRS, and EEG, employ distinct analytical methods making direct comparisons between studies challenging. For example, fMRI technology focuses on analyzing time series and spatial response patterns, while fNIRS calculates the coherence coefficients between time series for each dyad based on frequency and time, and EEG measures the phase locking of the EEG signals across time. To overcome these challenges and provide a more comprehensive understanding of neural similarity and synchrony among friends, future research should use multiple methods and conduct multi-faceted analyses to obtain converging evidence.

These future directions highlight the need for more sophisticated, precise, and varied research approaches to deepen our understanding of the neural underpinnings of friendship from the perspective of neural similarity and neural synchrony.

## Figures and Tables

**Table 1:** Summary of the literature about neural similarity and synchrony among friends.

Reference	Subjects	Techniques	Task	Independent Variables	Neural Synchrony or Similarity Index	Brain Regions	Specificity	Situational Modulations on INS
Baek et al., 2022 [8]	63 participants from a social network (1953 dyads)	fMRI successive scanning	Non-interaction (watching videos)	Individuals’ centrality within a social network	Temporal INS	Dorsomedial prefrontal cortex and precuneus	More central individuals > less central individuals (paired with all other members)	
Balconi and Fronda, 2020 [9]	15 friend dyads	fNIRS hyperscanning	Interaction (attentional cooperative task)	Before a gift exchange vs. after a gift exchange	Time-aligned temporal INS	Dorsolateral prefrontal cortex		Before a gift exchange < after a gift exchange
Balconi et al., 2020 [10]	14 friend dyads	EEG hyperscanning	Interaction (attentional cooperative task)	Before a gift exchange vs. after a gift exchange	Time-aligned temporal INS	Dorsolateral prefrontal cortex (delta and theta bands)		Before a gift exchange < after a gift exchange
Djalovski et al., 2021 [11]	34 friend dyads 52 stranger dyads (46 romantic partners)	EEG hyperscanning	Interaction (cooperative drawing; empathy giving task)	Friends vs. strangers	PLV	Motor task: central regions Empathy giving task: central and bilateral temporal regions	Friends = strangers	
Hyon et al., 2020a [12]	42 subjects from a social network (861 dyads)	fMRI successive scanning	Non-interaction (watching a series of videos)	Social distance within a social network	Spatial INS Spatio-temporal INS	Posterior parietal cortex	(1) Spatial INS: neural similarity is not correlated with social distance (2) Spatio-temporal INS: neural similarity decreases with increasing social distance	
Hyon et al., 2020b [13]	57 subjects from a social network (1596 dyads)	fMRI successive scanning	Non-interaction (rest)	Social distance within a social network	Functional connectomic INS	13 functional brain networks	Neural similarity decreases with increasing social distance	
Jia et al., 2024a [14]	26 friend dyads 26 stranger dyads (26 romantic partners)	EEG hyperscanning	Interaction (competitive button press task)	Friends vs. strangers	PLV	Frontal and occipital regions (Theta band)	Strangers > friends (400–600 ms time window); friends > strangers (800–1000 ms time window)	
Jia et al., 2024b [15]	12 friend dyads 10 stranger dyads	EEG hyperscanning	Interaction (motor imagery task)	No contact vs. eye contact vs. hand contact vs. eye and hand contact	CCorr	Frontal, parietal, and occipital; frontocentral regions		Theta band: eye and hand contact > other conditions; eye contact > no contact Alpha band: hand contact > no contact; eye and hand contact > other conditions
Liu et al., 2023 [16]	35 friend dyads	fNIRS hyperscanning	Interaction (interpersonal emotion regulation)	Cognitive reappraisal vs. expressive suppression	Time-aligned temporal INS	During the sharing stage: supramarginal gyrus and dorsolateral prefrontal cortex During the regulation stage: prefrontal cortex		During the sharing stage: cognitive reappraisal > expressive suppression During the regulation stage: cognitive reappraisal < expressive suppression
Long et al., 2020 [17]	22 friend dyads (22 romantic partners)	fNIRS hyperscanning	Interaction (free discussion)	Free discussion vs. touch	Time-lagged (2 s) WTC	Anterior temporal lobe in women and the temporoparietal junction and sensorimotor cortex in men		Free discussion > touch
Long et al., 2021 [18]	22 friend dyads (22 romantic partners)	fNIRS hyperscanning	Interaction (free discussion)	Supportive vs. conflictual vs. neutral topics	Time-lagged (4 s) WTC	Sensorimotor cortex		Supportive > conflictual Supportive > neutral
Luft et al., 2022 [19]	27 friend dyads 29 stranger dyads	EEG hyperscanning	Interaction (time reproduction task)	Friends vs. strangers Eye contact vs. no eye contact	PLV	Whole-brain network (Gamma band)	Friends > strangers	Eye contact > no eye contact
McNabb et al., 2020 [20]	68 subjects (school age; 767 dyads)	fMRI successive scanning	Non-interaction (rest)	Social distance within a social network	Functional connectomic INS	Whole-brain and default mode network, salience network, and bilateral fronto-parietal networks	Neural similarity is not correlated with social distance	
Pan et al., 2017 [21]	16 friend dyads 16 stranger dyads (17 romantic partners)	fNIRS hyperscanning	Interaction (cooperative button press task)	Friends vs. strangers	Time-aligned WTC	Right superior frontal cortex	Friends=strangers	
Parkinson et al., 2018 [22]	42 subjects from a social network (861 dyads)	fMRI successive scanning	Non-interaction (watching videos)	Social distance within a social network	Temporal INS	Superior and inferior parietal lobe, nucleus accumbens, amygdala, putamen, and caudate nucleus.	Neural similarity decreases with increasing social distance	
Song et al., 2024 [23]	23 friend dyads 24 stranger dyads	fNIRS hyperscanning	Interaction (joint Simon task)	Friends vs. strangers	Time-aligned temporal INS	Dorsolateral and medial parts of the prefrontal cortex	Friends > strangers	
Spiegelhalder et al., 2014 [24]	11 friend dyads	fMRI hyperscanning	Interaction (discussions)	Real conversational friends vs. not actually conversational dyads	Temporal INS	Speech production areas synchronized with auditory cortex and posterior cingulate cortex		Real conversational friends > not actually conversational dyads
Zhang et al., 2024 [25]	18 friend dyads 18 stranger dyads	fNIRS hyperscanning	Interaction (tangram puzzle task)	Friends vs. strangers Collaborative cooperation vs. division of labor cooperation	Time-aligned WTC	Specificity: bilateral dorsolateral prefrontal cortex and the right temporoparietal junction Situational modulations left dorsolateral prefrontal cortex, right pars triangularis, and right supramarginal gyrus	Friends = strangers	Collaborative cooperation > division of labor cooperation
Zhao et al., 2023 [26]	30 friend dyads 30 stranger dyads	fNIRS hyperscanning	Interaction (Balloon Analogue Risk Task)	Friend dyads vs. stranger dyads Negative feedback vs. positive feedback	Time-aligned WTC	Right dorsolateral prefrontal cortex, bilateral inferior prefrontal gyrus, and frontopolar cortex		Friend dyads: negative feedback > positive feedback

## References

[1] Byrne D., Gouaux C., Griffitt W., Lamberth J., Murakawa N., Prasad M., Prasad A., Ramirez M. (1971). The ubiquitous relationship: Attitude similarity and attraction. Hum. Relations.

[2] McPherson M., Smith-Lovin L., Cook J.M. (2001). Birds of a feather: Homophily in social networks. Annu. Rev. Sociol..

[3] Feiler D.C., Kleinbaum A.M. (2015). Popularity, Similarity, and the Network Extraversion Bias. Psychol. Sci..

[4] Winslow C.N. (1937). A study of the extent of agreement between friends’ opinions and their ability to estimate the opinions of each other. J. Soc. Psychol..

[5] Han X., Wang L., Crespi N., Park S., Cuevas Á. (2015). Alike people, alike interests? Inferring interest similarity in online social networks. Decis. Support Syst..

[6] Christakis N.A., Fowler J.H. (2014). Friendship and natural selection. Proc. Natl. Acad. Sci. USA.

[7] Brent L.J., Chang S.W., Gariépy J., Platt M.L. (2013). The neuroethology of friendship. Ann. N. Y. Acad. Sci..

[8] Baek E.C., Hyon R., López K., Finn E.S., Porter M.A., Parkinson C. (2022). In-degree centrality in a social network is linked to coordinated neural activity. Nat. Commun..

[9] Balconi M., Fronda G. (2020). The "gift effect" on functional brain connectivity. Inter-brain synchronization when prosocial behavior is in action. Sci. Rep..

[10] Balconi M., Fronda G., Vanutelli M.E. (2020). When gratitude and cooperation between friends affect inter-brain connectivity for EEG. BMC Neurosci..

[11] Djalovski A., Dumas G., Kinreich S., Feldman R. (2021). Human attachments shape interbrain synchrony toward efficient performance of social goals. NeuroImage.

[12] Hyon R., Kleinbaum A.M., Parkinson C. (2020). Social network proximity predicts similar trajectories of psychological states: Evidence from multi-voxel spatiotemporal dynamics. NeuroImage.

[13] Hyon R., Youm Y., Kim J., Chey J., Kwak S., Parkinson C. (2020). Similarity in functional brain connectivity at rest predicts interpersonal closeness in the social network of an entire village. Proc. Natl. Acad. Sci. USA.

[14] Jia S., Meng Y., Gao Y., Ao L., Yang L., Wang H., Liu Y. (2024). Romantic relationships attenuated competition between lovers: Evidence from brain synchronization. Cereb. Cortex.

[15] Jia T., Sun J., McGeady C., Ji L., Li C. (2024). Enhancing brain–computer interface performance by incorporating brain-to-brain coupling. Cyborg Bionic Syst..

[16] Liu Z., Lu K., Hao N., Wang Y. (2023). Cognitive reappraisal and expressive suppression evoke distinct neural connections during interpersonal emotion regulation. J. Neurosci..

[17] Long Y., Zheng L., Zhao H., Zhou S., Zhai Y., Lu C. (2020). Interpersonal neural synchronization during interpersonal touch underlies Affiliative pair bonding between romantic couples. Cereb. Cortex.

[18] Long Y., Chen C., Wu K., Zhou S., Zhou F., Zheng L., Zhao H., Zhai Y., Lu C. (2021). Interpersonal conflict increases interpersonal neural synchronization in romantic couples. Cereb. Cortex.

[19] Luft C.D.B., Zioga I., Giannopoulos A., Di Bona G., Binetti N., Civilini A., Latora V., Mareschal I. (2022). Social synchronization of brain activity increases during eye-contact. Commun. Biol..

[20] McNabb C.B., Burgess L.G., Fancourt A., Mulligan N., FitzGibbon L., Riddell P., Murayama K. (2020). No evidence for a relationship between social closeness and similarity in resting-state functional brain connectivity in schoolchildren. Sci. Rep..

[21] Pan Y., Cheng X., Zhang Z., Li X., Hu Y. (2017). Cooperation in lovers: An fNIRS-based hyperscanning study. Hum. Brain Mapp..

[22] Parkinson C., Kleinbaum A.M., Wheatley T. (2018). Similar neural responses predict friendship. Nat. Commun..

[23] Song X., Dong M., Feng K., Li J., Hu X., Liu T. (2024). Influence of interpersonal distance on collaborative performance in the joint Simon task—An fNIRS-based hyperscanning study. NeuroImage.

[24] Spiegelhalder K., Ohlendorf S., Regen W., Feige B., Van Elst L.T., Weiller C., Hennig J., Berger M., Tüscher O. (2014). Interindividual synchronization of brain activity during live verbal communication. Behav. Brain Res..

[25] Zhang M., Yin Z., Zhang X., Zhang H., Bao M., Xuan B. (2024). Neural mechanisms distinguishing two types of cooperative problem-solving approaches: An fNIRS hyperscanning study. NeuroImage.

[26] Zhao H., Zhang C., Tao R., Duan H., Xu S. (2023). Distinct inter-brain synchronization patterns underlying group decision-making under uncertainty with partners in different interpersonal relationships. NeuroImage.

[27] Li B., Cheng T., Guo Z. (2021). A review of EEG acquisition, processing and application. J. Phys. Conf. Ser..

[28] Ferrari M., Quaresima V. (2012). A brief review on the history of human functional near-infrared spectroscopy (fNIRS) development and fields of application. NeuroImage.

[29] Geethanath S., Vaughan J.T. (2019). Accessible magnetic resonance imaging: A review. J. Magn. Reson. Imaging.

[30] Burle B., Spieser L., Roger C., Casini L., Hasbroucq T., Vidal F. (2015). Spatial and temporal resolutions of EEG: Is it really black and white? A scalp current density view. Int. J. Psychophysiol..

[31] Scarapicchia V., Brown C., Mayo C., Gawryluk J.R. (2017). Functional magnetic resonance imaging and functional near-infrared spectroscopy: Insights from combined recording studies. Front. Hum. Neurosci..

[32] Nastase S.A., Gazzola V., Hasson U., Keysers C. (2019). Measuring shared responses across subjects using intersubject correlation. Soc. Cogn. Affect. Neurosci..

[33] Léné P., Karran A.J., Labonté-Lemoyne E., Sénécal S., Fredette M., Johnson K.J., Léger P. (2019). Wavelet transform coherence: An innovative method to investigate social interaction in NeuroIS. Information Systems and Neuroscience.

[34] Nam C.S., Choo S., Huang J., Park J. (2020). Brain-to-brain neural synchrony during social interactions: A systematic review on Hyperscanning studies. Appl. Sci..

[35] Jiang J., Zheng L., Lu C. (2020). A hierarchical model for interpersonal verbal communication. Soc. Cogn. Affect. Neurosci..

[36] Burgess A.P. (2013). On the interpretation of synchronization in EEG hyperscanning studies: A cautionary note. Front. Hum. Neurosci..

[37] Cui X., Bryant D.M., Reiss A.L. (2012). NIRS-based hyperscanning reveals increased interpersonal coherence in superior frontal cortex during cooperation. NeuroImage.

